# Perimyocarditis Associated with Immune Checkpoint Inhibitors: A Case Report and Review of the Literature

**DOI:** 10.3390/medicina60020224

**Published:** 2024-01-28

**Authors:** Walid Shalata, Rachel Steckbeck, Amjad Abu Salman, Omar Abu Saleh, Ashraf Abu Jama, Zoé Gabrielle Attal, Sondos Shalata, Hilmi Alnsasra, Alexander Yakobson

**Affiliations:** 1 The Legacy Heritage Oncology Center and Dr. Larry Norton Institute, Soroka Medical Center & Ben-Gurion University, Beer-Sheva 84105, Israel; 2 Medical School for International Health and Sciences, Ben-Gurion University, Beer-Sheva 84105, Israel; 3 Cardiology Department, Soroka Medical Center & Ben-Gurion University, Beer-Sheva 84105, Israel; 4 Department of Dermatology and Venereology, Emek Medical Centre, Afula 18341, Israel; 5 Nutrition Unit, Galilee Medical Center, Nahariya 22000, Israel

**Keywords:** immune checkpoint inhibitor (ICIs), lung cancer, Yervoy^®^ (ipilimumab), Opdivo^®^ (nivolumab), perimyocarditis, myocarditis, pericarditis

## Abstract

Patient prognoses have been significantly enhanced by immune checkpoint inhibitors (ICIs), altering the standard of care in cancer treatment. These novel antibodies have become a mainstay of care for metastatic non-small-cell lung cancer (mNSCLC) patients. Several types of adverse events related to ICIs have been identified and documented as a result of the launch of these innovative medicines. We present here a 74-year-old female patient with a stage IV lung adenocarcinoma, treated with nivolumab plus ipilimumab, who developed perimyocarditis two weeks after receiving the third cycle of immune checkpoint inhibitor therapy. The patient was diagnosed using troponin levels, computed tomography (CT) angiography, and echocardiography. After hospitalization, her cardiac condition was successfully resolved with corticosteroids, colchicine, and symptomatic treatment. To the best of our knowledge, this is one of the rarest cases to be reported of perimyocarditis as a toxicity of immunotherapy in a patient treated for adenocarcinoma of the lung.

## 1. Introduction

Lung cancer remains the cause of the most cancer-related deaths in the United States and is a major healthcare concern throughout the world [1]. Non-small-cell lung cancer (NSCLC) accounts for nearly 85% of all lung cancer diagnoses. Most patients are at an advanced stage of disease at the time of diagnosis, presenting a poor prognosis [2]. 

Immune checkpoint inhibitors have been the backbone of treatment for metastatic lung cancer in recent years. Monoclonal antibodies against programmed death-1 (PD-1) and its ligands, as well as cytotoxic T-lymphocyte-associated antigen-4 (CTLA-4), all of which have complementary modes of action, are among them. Nivolumab is an IgG4 antibody that targets the protein PD-1 on T lymphocyte surfaces; it prevents PD-1 from interacting with its ligands, allowing T lymphocytes to detect and destroy tumor cells without inhibition. Ipilimumab, on the other hand, is a humanized anti-cytotoxic T-lymphocyte antigen-4 (CTLA-4) antibody. It works similarly to nivolumab in that it blocks the inhibition of T cells, thus allowing them to attack tumor cells. The findings of two separate studies, CheckMate 227 and Checkmate 9LA, support nivolumab plus ipilimumab as a treatment in first-line therapy for advanced NSCLC regardless of the PD-L1 expression level [3]. The side effects of nivolumab and other immune checkpoint inhibitors are not the same as those of cytotoxic chemotherapy. Because immune checkpoint inhibitors activate T cells, the majority of their side effects are immunologically mediated, including skin rash, pericarditis, thyroiditis, colitis, hepatitis, pneumonitis, and hypophysitis [4]. These side effects often occur within a year after starting ICI treatment [3,4]. Cardiac complications are very rare immune-related side effects of ICIs, occurring in less than 1% of patients; however, these complications have a mortality rate of up to 50% [5].

The clinical diagnosis of perimyocarditis, denoting predominant pericarditis with myocardial involvement, is established when patients meet definite criteria for acute pericarditis and display elevated biomarkers of myocardial injury (troponin I or T, CK-MB fraction). This diagnosis is made without the presence of newly developed focal or diffuse impairment of left ventricular function observed in echocardiography or CMR. Perimyocarditis, as a term, characterizes a primarily pericarditic syndrome with minor myocardial involvement, representing the majority of combined pericarditis and myocarditis cases seen in clinical practice [6,7,8,9,10].

Conversely, if patients with elevated myocardial biomarkers and clinical criteria for acute pericarditis exhibit evidence of new-onset focal or diffuse reduction of left ventricular function, it suggests predominant myocarditis with pericardial involvement, termed “perimyocarditis.” Definitive confirmation of myocarditis requires an endomyocardial biopsy, according to the Myocardial and Pericardial Diseases Working Group position statement [6,7,8,9,10,11,12,13]. 

However, in cases where patients with suspected concomitant myocardial involvement in predominant pericarditis (perimyocarditis) show absent or mild left ventricular dysfunction and no symptoms of heart failure, the favorable prognosis does not necessitate relying on endomyocardial biopsy as a clinical requirement. Therefore, knowledge of ICI cardiotoxicity is crucial for clinicians treating patients with ICIs [6,7,8,9,10,11,12,13]. In the current article, the patient developed both pericarditis and myocarditis upon treatment of NSCLC with immune checkpoint inhibitors. 

## 2. Case Study

**Figure 1:**
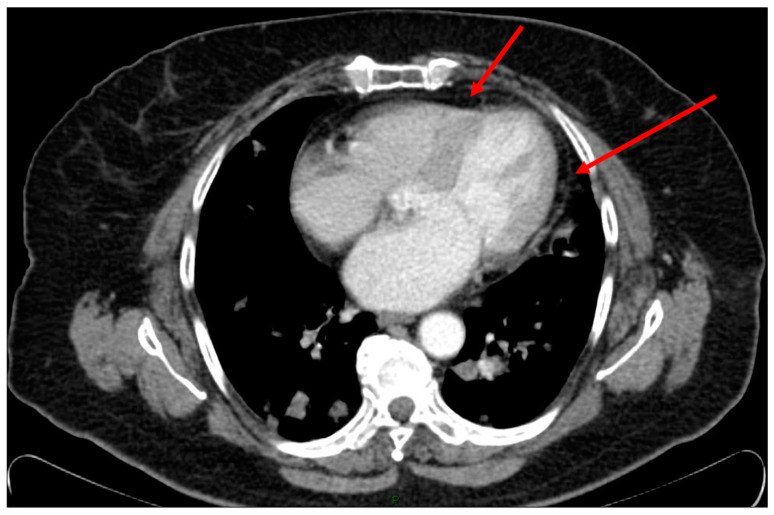
Computed tomography angiography of the chest at presentation, showing the pericardial effusion (the black area near the red arrow).

A 74-year-old female without a history of cigarette smoking was diagnosed with metastatic lung adenocarcinoma in both lobes of the lung (stage 4). Molecular testing was negative for EGFR-, ALK-, and ROS-, and PD-L1 was less than 1%. The patient started treatment in July 2020, in which she received three cycles of nivolumab (3 mg per kg every 3 weeks) plus ipilimumab (1 mg per kg every 6 weeks) as first-line therapy. The third cycle was in November 2020, and her last echocardiography from March 2019 showed a left ventricular ejection fraction of 65%. In December 2020 (two weeks after receiving the third cycle), the patient presented to the emergency department with exacerbated fatigue and palpitations. During her stay in the emergency room, she exhibited a decrease in her general condition alongside hemodynamic deterioration (fever of 38.7 °C, hypotension of 70/39, oxygen saturation of 90%) and tachycardia of 157 bpm. On physical examination, heart sounds were normal without any audible murmur or rubs, the lung was clear to auscultation, and an abdominal examination demonstrated no hepatosplenomegaly. The electrocardiogram (ECG) showed diffuse elevation of the ST segment without any reciprocal changes. A complete blood count and chemistry showed leukocytes, neutrophils, and platelets in normal ranges. C-reactive protein was 13 mg/dL (normal range 0.02–0.05 mg/dL) and troponin I was 273 ng/L (normal range 0–14 ng/L). We completed a computed tomography angiogram of the chest due to a suspicion of pulmonary embolism; no filling defects in the pulmonary arteries were demonstrated, but a small pleural effusion on the right, pericardial effusion, and secondary bilateral pulmonary scattering were detected in an exacerbation (Figure 1). She also underwent an echocardiography imaging that demonstrated a small pericardial effusion, preserved LV function, an LV ejection fraction of 50%, and mild tricuspid regurgitation without any wall motion abnormality. A second troponin test showed a decrease in her troponin level to 171 ng/L.

After stabilizing her condition (she was treated with antipyretics, fluids, and norepinephrine), the patient was admitted to the department of internal medicine for further clarification and treatment. In the internal department, viral serology and blood culture were negative, and the rheumatology panel was at a normal level.

Due to the presence of diffuse ST elevations, a new pericardial effusion, and increased troponin without other heat sources or signs of ischemic cardiomyopathy, she was diagnosed with acute perimyocarditis. A multidisciplinary conference including a cardiologist, oncologist, and infectious disease specialist concluded that the clinical presentation of the patient was consistent with acute perimyocarditis as a toxicity of immunotherapy. The therapy of nivolumab plus ipilimumab was discontinued. In cardiology counseling, it was decided that she, at this stage, did not need a coronary diagnostic clarification. We initiated a conservative treatment with high-dose prednisone (2 mg/kg) and colchicine. After a few days, the patient demonstrated an excellent clinical response, therefore, dosage tapering down started (5 mg every 5 days). Two weeks later, she was admitted for follow-up. At that time, she reported none of the previous symptoms, had no other complications, and her ECG showed normal ranges of waves (ST). Due to these immune-related adverse events (irAEs), immunotherapy (nivolumab and ipilimumab) was stopped and she continued receiving only chemotherapy (carboplatin and pemetrexed) at the same previous dosage. The patient died due to disease progression 3 months later.

## 3. Diagnosis

ICI-induced myocarditis is typically associated with elevated troponin, cardiac serum markers, and abnormal echocardiogram (ECG) findings. As such, a troponin test and ECG should be performed if symptoms of myocarditis are present. 

In myocarditis, ECGs can show a range of changes including: non-specific T wave changes, ST elevation myocardial infarction (STEMI), sinus tachycardia, the presence of Q waves, ventricular arrhythmias, etc. [14,15,16,17,18]. ECGs are abnormal in 89% of myocarditis cases; however, with a sensitivity nearing 47%, they are insufficient to pose a diagnosis. Similarly, troponin sensitivity and specificity are low; however, it is a requirement for the diagnosis of ICI-related myocarditis. Additionally, troponin has an impact on treatment due to its prognostic abilities (i.e., higher troponin is suggestive of worse outcomes) [17]. Symptomatic patients should have cardiac serum markers such as BNP and NT-proBNP measured to detect cardiac strain [15]. 

Amongst first-line examinations, if ICI-induced myocarditis is suspected, an echocardiography is routinely performed. If the examination is normal, further tests such as coronary angiography or cardiac stress tests can be performed if the threat of adverse cardiac events remains high [14,15].

Magnetic resonance imaging (MRI) is equally to be considered. Debate has surrounded their use in the context of myocarditis. However, recent studies demonstrate their utility in the diagnosis of ICI-induced myocarditis. T1- and T2-weighted images, and gadolinium enhancement can detect myocarditis because of the increased presence of fluids and necrosed materials found in hearts caused by increased capillary permeability. MRI waitlists and access prevent the routine use of this imagery tool regardless of a high suspicion of myocarditis. It is essential to note that negative results in MRIs, ECG, or echocardiography do not definitively reject the possibility of myocarditis [14,15,16,17]. 

According to the American Heart Association (AHA) guidelines, an endomyocardial biopsy (EMB) is the gold standard practice. Due to its invasiveness, its risks can tend to outweigh the benefit they provide regarding confirmation of diagnosis. Additionally, EMB has low sensitivity and requires multiple samples, increasing the burden of the procedure [18]. 

The AHA also recommends testing for a wide array of underlying causes of myocarditis. Some of the possible causes are virus- and immune-mediated lymphocytic myocarditis, eosinophilic or giant cell myocarditis, and sarcoidotic myocarditis. Once ICI myocarditis is identified and treatment is initiated, guidelines suggest checking the patient’s troponin level weekly for 6 weeks [14,15,16,17,18].

## 4. Management and Treatment

Cardiac immune-related adverse events (irAEs) associated with immune checkpoint inhibitors (ICIs) are rare but life-threatening complications. The Society for Immunotherapy of Cancer divides them into four grades:

Grade I—defined as largely asymptomatic patients, requiring no treatment or arrest of ICI therapy. Tests show abnormal cardiac biomarkers or ECG. 

Grade II—patients have minimal symptoms that tend to resolve with halting immunotherapy, and have abnormal cardiac biomarkers and ECG. 

Grade III—cardiac symptoms are consequential, and ICI therapy is to be halted urgently. TTE shows LVEF < 50% or regional wall motion cardiac MRI is diagnostic or suggestive of myocarditis. 

Grade IV—cardiac irAEs are life threatening with cardiac testing abnormalities from Grade I to III [14,15,16,17,18,19,20,21,22]. 

Treatment depends on the grade of myocarditis and is largely supportive. In lower grade (I–II) myocarditis, suspending treatment temporarily is recommended. In severe grade IV myocarditis, ICIs are halted, and the patient is given high-dose intravenous (IV.) corticosteroids as first-line therapy (prednisone 1–2 mg/kg/d or methylprednisolone 1–2 mg/kg/d). In severe myocarditis, IV. immunoglobulin (IV.IG), anti-thymocyte globulin (anti-CD3 antibody), alemtuzumab (anti-CD52 antibody), and abatacept (CTLA4 agonist) can be administered as second-line therapy. In grades I–IV, close cardiac monitoring is essential to prevent the recurrence of myocarditis [16,17,18,19,20,21,22,23,24,25,26,27,28,29,30,31,32,33,34,35,36,37,38,39].

## 5. Types of Cardiac Toxicity Associated with Immune Checkpoint Inhibitors 

It’s crucial to observe that males and individuals of African descent face a greater risk of experiencing cardiac adverse events in comparison to females and those of Caucasian ethnicity, respectively [14,15,40,41].

### 5.1. Pericarditis

Pericarditis (pericardial disease), an inflammation involving the sac enveloping the heart, is another prevalent form of cardiotoxicity, occurring in about 0.3% of cases. Although its fatality is less than that of myocarditis, it still poses a considerable risk, with mortality rates ranging from 13% to 21%. Typically, patients develop pericarditis within the first 30 days of commencing immune checkpoint inhibitor (ICI) treatment, presenting with symptoms such as chest pain, shortness of breath, and hemodynamic instability. Among these, shortness of breath emerges as the most commonly reported symptom. Notably, studies suggest a stronger correlation between pericardial disease and the use of anti-PD-1 or anti-PD-L1 therapies compared to anti-CTLA-4 therapy [14,15,40,41,42,43,44,45].

### 5.2. Myocarditis

Myocarditis, characterized by inflammation of the myocardial muscle, stands out as the most prevalent form of cardiotoxicity induced by immune checkpoint inhibitors (ICIs). Interestingly, it is also the most lethal, with a mortality rate reaching up to 50%. Initial recognition of myocarditis as an immune-related adverse event (IRAE) occurred during clinical trials. The onset of myocarditis can occur as early as 2 weeks after initiating ICI therapy. However, the median time for patients to experience symptoms is around 65 days, with 81% of them presenting within 3 months of starting therapy. Commonly, reported symptoms include shortness of breath, palpitations, and indications of congestive heart failure, such as edema, fatigue, weakness, or wheezing. Laboratory tests often reveal elevated levels of BNP or NT-proBNP, signs of active inflammation such as increased CRP and hepcidin, and elevated troponin and CK-MB. The gold standard for diagnosing myocarditis is an endomyocardial biopsy. It is noteworthy that patients with myocarditis often exhibit high expression of PD-L1 in cardiac tissue [14,15,40,41,42,43,44,45].

### 5.3. Arrhythmias

Conduction disorders induced by immune checkpoint inhibitors encompass atrial fibrillation, ventricular tachycardia or fibrillation, and atrioventricular conduction disorders. These conditions may manifest independently or in tandem with myocarditis. Notably, all conduction diseases are linked to heightened mortality. The precise etiology of conduction diseases remains uncertain, with suggestions pointing towards local inflammation or fibrosis as potential contributors. Some argue that systemic inflammation triggered by cancer and ICI treatment could exacerbate cardiovascular conditions, potentially culminating in arrhythmias [14,15,40,41,42,43,44,45].

### 5.4. Takotsubo Cardiomyopathy

Takotsubo cardiomyopathy represents a unique manifestation of stress-induced cardiomyopathy and is an infrequent occurrence associated with immune checkpoint inhibitor (ICI)-induced cardiotoxicity. Typically, patients begin experiencing symptoms of Takotsubo cardiomyopathy between 15 weeks and 8 months into their ICI treatment. The origin of this condition is not fully understood, and research in this specific presentation is limited due to its relative rarity. Potential causes of Takotsubo cardiomyopathy in the context of ICI-associated cardiotoxicity include a direct impact of ICIs on the heart. Alternatively, it may be triggered by a sudden surge of catecholamines. There is also speculation that it could result from delayed cardiotoxicity stemming from previous rounds of chemotherapy, particularly in cases where ICIs were not the initial line of therapy [14,15,40,41,42,43,44,45].

## 6. Types of Immune Checkpoint Inhibitors Associated with Cardiac Toxicity

### 6.1. PD-1 Inhibitors

#### 6.1.1. Pembrolizumab

Pembrolizumab is a humanized IgG4 antibody targeting PD-1. Unlike typical IgG antibodies, pembrolizumab does not induce antibody-dependent cellular cytotoxicity. Instead, it predominantly leads to immune-related adverse events, observed in approximately 70% of patients. While the drug is known for its immunomodulatory effects, cardiotoxicity, such as myocarditis and pericarditis, is a rare occurrence, affecting less than 1% of patients. Initially approved by the FDA in 2014, pembrolizumab has gained approval for the treatment of various cancers and is actively used in clinical settings [14,46,47,48,49,50].

#### 6.1.2. Nivolumab

Nivolumab is a fully human IgG4 antibody that targets PD-1 and operates independently of the antibody-dependent cellular cytotoxicity pathway. While cardiac adverse effects are observed in less than 1% of patients, it is noteworthy that the combination therapy of ipilimumab and nivolumab shows a higher incidence of cardiac adverse effects compared to either treatment alone. Despite the rarity of cardiotoxicity, caution is warranted, especially in combined therapy regimens. Initially approved by the FDA in 2014, nivolumab has garnered approval for the treatment of a wide range of cancers [14,46,47,48,51,52].

#### 6.1.3. Cemiplimab

Cemiplimab, a humanized IgG4 monoclonal antibody designed to selectively target PD-1, shares a similar mechanism of action with fellow drugs nivolumab and pembrolizumab. It has gained FDA approval for treating various types of cancer. There have been no prior reports associating cardiotoxicity with cemiplimab [14,46,47,48,53].

### 6.2. Anti CTLA-4

#### Ipilimumab

Ipilimumab is a fully human recombinant antibody designed to target CTLA-4. Its unique mechanism of action involves stimulating the immune system, which distinguishes it from traditional cytotoxic chemotherapy. This differentiation results in patients typically not experiencing common chemotherapy side effects such as bone marrow suppression. However, up to 90% of patients may develop immune-related adverse events due to the immunostimulatory nature of ipilimumab. Cardiotoxicity associated with ipilimumab is exceptionally rare, occurring in less than 1% of patients. The drug has demonstrated efficacy and safety in the treatment of various cancers. Approved by the FDA in 2011, ipilimumab has since become a valuable therapeutic option for multiple cancer types [14,46,47,48,54,55].

### 6.3. PDL-1 Inhibitors

#### 6.3.1. Durvalumab

Durvalumab, a fully human IgG antibody targeting PD-L1, is approved for the treatment of several types of cancers. Notably, cardiotoxicity associated with this drug is exceedingly rare, with only three reported cases. Durvalumab has demonstrated efficacy and safety in the treatment of cancer. Initially approved by the FDA in 2017, durvalumab continues to be a valuable therapeutic option in the field of oncology, providing a targeted approach against PD-L1 to enhance the body’s immune response against cancer cells [14,36,46,47,48,56,57,58,59].

#### 6.3.2. Atezolizumab

Atezolizumab is a PD-L1 inhibitor approved by the FDA for the treatment of various types of cancer. The incidence of cardiotoxicity with atezolizumab is reported to be less than 1%, and only one case has been specifically reported in the literature [14,46,47,48,60,61].

#### 6.3.3. Avelumab

Avelumab, a PD-L1 inhibitor, has received FDA approval for treating various types of cancer. Importantly, there have been no previous reports associating avelumab with cardiac toxicity [14,46,47,48,62,63].

## 7. Screening and Surveillance

In cases where there is a heightened risk of cardiotoxicity due to cancer treatments, it is imperative to institute comprehensive screening and surveillance protocols. Specifically, before commencing anthracycline therapy, evaluating the left ventricular ejection fraction (LVEF) is recommended. However, for immune checkpoint inhibitor (ICI) therapy initiation, pre-treatment LVEF assessment may lack utility, given that data indicates 70% of individuals developing myocarditis during ICI therapy had normal LVEF levels before treatment onset. The majority of studies suggest the presence of abnormal electrocardiograms (ECGs) and elevated cardiac troponin (cTn) levels at the onset of symptoms. Consequently, considering serial ECG and cTn monitoring for surveillance purposes becomes pertinent. Given the relatively short median time to myocarditis, measuring cTn levels at the initiation and conclusion of each cycle could prove beneficial. In case of an elevated cTn level indicative of myocarditis, immediate consultation with cardiology or cardio-oncology for further assessment is crucial. Other critical considerations involve defining the patient population requiring monitoring and determining the duration of surveillance. Patients undergoing therapy for adjuvant, neoadjuvant or for metastatic disease, those utilizing combination ICI regimens, and individuals receiving ICIs alongside other treatments recognized for their cardiovascular toxicities are potential candidates who may derive substantial benefits from vigilant surveillance [35,64,65,66,67,68,69,70,71,72,73,74,75].

## 8. Discussion 

Cardiotoxicities due to ICIs are rare. Some researchers have reported myocarditis incidence to be as high as 1% [5,17,18,19,20,21,22,23]. However, others state that there may be many other cardiac events happening in patients taking ICI treatment that are not symptomatic and thus not recorded. 

As in our patient, myocarditis or inflammation of the myocardial muscle is the most common form of cardiotoxicity caused by ICIs. As mentioned before, it is the most fatal form of cardiotoxicity, with a mortality rate of up to 50% [76,77,78,79,80,81,82,83,84].

Patients can have a wide range of signs and symptoms, from asymptomatic to severe chest pain, dyspnea, and multi-organ failure, and sudden death. These symptoms usually begin within the first 3 months of starting immunotherapy, but they can also start up to a year after therapy finishes [34,36,37,38,39,81,82,83,84,85,86].

**Figure 2:**
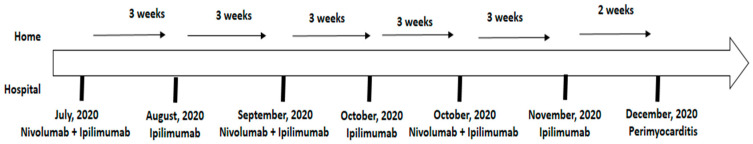
The patient’s timeline for cancer treatment and the nivolumab-plus-ipilimumab-induced adverse effect, perimyocarditis.

This case is unusual in that the patient’s cardiotoxicity presented two weeks after receiving the third cycle of treatment (Figure 2); normally, time from onset of treatment to presentation of cardiotoxicity is 2–3 months, and patients will often present after only 1 or 2 rounds of treatment [14,21,22,23,24,25,26,27,28].

This case is also unusual in that the patient presented with pericarditis and myocarditis concurrently. After an extensive literature search, we did not find any previous case of perimyocarditis as a toxicity of immunotherapy; our patient is the first to be reported.

Cardiac toxicity as a result of ICI treatment can be diagnosed in a number of ways. In the current case, the patient was diagnosed using troponin levels, CT angiography, and an echocardiogram. The gold standard of cardiotoxicity diagnosis is an endomyocardial biopsy; however, it is an invasive procedure, so it is not used unless the diagnostic benefits outweigh the substantial risks [15,16,17,18,19,20,21,22,23,24,25,26,27,28,29,30,31,32,33,34,35,36]. Other methods of diagnosis include an EKG, CMR, and NT-proBNP [14,15,16,17,18,19,20,21,22,23,24,25,26,27,28,29,30,31,32,33,34,35,36,37,38,39,69,70,71,72,73].

For the best outcome after presentation with cardiac irAEs, the American Society of Clinical Oncology clinical practice guidelines for the management of irAEs in patients treated with ICI therapy recommend high-dose corticosteroids (1 to 2 mg/kg prednisone) for high-grade cardiovascular irAEs, and management should be multidisciplinary, with the involvement of a cardiologist or cardio-oncologist and an immunity oncologist [5,14,15,16,17,18,19,21,22,23,24,29,30,31,32,33,34,35,36,37,38,39].

In accordance with this, the patient was treated with high-dose corticosteroids, in addition to colchicine and symptomatic treatment. With this treatment, the patient’s cardiotoxicity resolved. 

It is necessary for both the oncologist and cardiologist to have a high suspicion of ICI-related cardiac toxicity. Studies evaluating multimodality imaging and invasive testing with endomyocardial biopsy will guide development of better diagnostic algorithms for this condition. Current treatment is largely based on glucocorticoids with a possible role for more targeted immune modulators, depending on the clinical course of individual patients [39,77,78,79,80,81,82,83,84,85]. The European Society of Cardiology guidelines for cancer therapy-related CV toxicity recommend that the timing and frequency of monitoring after initial assessment should be individualized based on the patient’s baseline CV risk and the chemotherapy protocol; however, there is no mention of strategies for prevention and management of ICI-induced cardiotoxicity [19]. Myocarditis can often be misdiagnosed as ST-elevation myocardial infarction (STEMI) during the initial presentation due to various reasons. Firstly, the symptoms of myocarditis exhibit considerable variability and can significantly overlap with those of a myocardial infarction. Common symptoms include dyspnea, chest pain, palpitations, fever, lower extremity edema, fatigue, and syncope. However, as seen in this particular case, patients might only experience acute chest pain without additional symptoms. Moreover, certain indicators in a patient’s history that could guide a physician to the correct diagnosis might be either too sensitive for the patient to discuss, such as a history of drug use or HIV, or may not register as noteworthy to the patient, including recent flu-like symptoms or vaccinations. Therefore, it is incumbent upon the physician to gather a comprehensive history, probing for information specific to the potential diagnosis [85,86,87].

An analysis of a recent case series revealed that patients with myocarditis mimicking STEMI typically share characteristics, such as a young age, a preceding infectious prodrome, and elevated inflammatory biomarkers. Through identifying which patients are at higher risk for myocarditis, healthcare providers can make informed decisions about further testing before resorting to invasive interventions like an endomyocardial biopsy. Additionally, cardiac MRI (CMR) offers a non-invasive means of tissue characterization for the myocardium, aiding in the diagnosis of myocarditis. While distinguishing between myocarditis and STEMI can be challenging in some scenarios, a combination of CMR, comprehensive history-taking, and adherence to diagnostic criteria for clinically suspected myocarditis can collectively heighten the suspicion of myocarditis [85,86,87].

Both myocarditis and arrhythmogenic right ventricular dysplasia can be effectively examined using CMR. A recent consensus has outlined specific criteria for diagnosing myocarditis, with CMR playing a dominant role. According to this consensus, a diagnosis of myocarditis can be established when at least two of the following features are present: the presence of non-ischemic late gadolinium enhancement in one area, global or regional signal increase in T2-weighted images, and global early gadolinium enhancement. Additionally, CMR studies can offer valuable insights into morphological and functional aspects, contributing to a more comprehensive investigative approach [87,88,89,90].

Our case review has a few limitations. First, the sample size is small. Second, myocardial biopsy and pericardial drainage were not performed in this patient to confirm the diagnosis or completely exclude a malignant cause. However, the disease’s course and the quick recovery of the pericardial effusion make a malignant cause less likely.

In the future, more research needs to be carried out on the recurrence of ICI-associated cardiotoxicity such as randomized trial investigation. In addition, investigation is needed into the incidence of ICI-associated cardiotoxicity in women versus men, since women generally have a higher risk of autoimmune diseases and are underrepresented in clinical trials due to this fact [14,19,35,39,63,65,82,83,84,91,92,93]. As we have searched in the literature, we have found only two cases reported about myo- and pericarditis as complications of immune checkpoint inhibitors [94,95]. Hence, our case underscores the importance of considering and understanding the potential complications associated with immune checkpoint inhibitors, even though they may be infrequent.

## 9. Conclusions

Immune checkpoint inhibitors may cause various immune-related side effects, including the rare yet serious occurrence of cardiotoxicity. Despite their uncommon nature, these side effects carry a high risk of mortality. Thus, it is crucial to stay alert in recognizing common symptoms and relevant lab results during patient treatment. Within this article, we explore a case of exceedingly uncommon cardiotoxicity linked to these inhibitors. This report emphasizes the necessity for vigilant monitoring, recognizing and managing, and prompt intervention to manage ICI-induced adverse events for optimized patient care and treatment outcomes.

## Data Availability

The data either resides within the article itself or can be obtained from the authors upon making a reasonable request.
